# Barriers and facilitators of access to maternity care for African-born women living in Australia: a meta-synthesis of qualitative evidence

**DOI:** 10.1186/s13643-024-02628-8

**Published:** 2024-08-09

**Authors:** Ayele Geleto Bali, Vidanka Vasilevski, Linda Sweet

**Affiliations:** 1 School of Nursing and Midwifery, Deakin University, Burwood, VIC Australia; 2 Centre for Quality and Patient Safety Research, Institute for Health Transformation, Western Health Partnership, Melbourne, VIC Australia

**Keywords:** Meta-synthesis, Maternity, Barriers, Facilitators, Africa-born women, Australia

## Abstract

**Background:**

Adverse perinatal health outcomes are notably high among African-born women living in Australia. This problem is partly attributed to their lower engagement in maternity care services as compared to Australian-born women. Various barriers might limit African-born women’s access to and use of services; however, these barriers are not well documented. Therefore, this review aimed to synthesise current qualitative evidence on barriers and facilitators of access to maternity care for African-born women living in Australia.

**Methods:**

The search was conducted in MEDLINE, CINAHL, Embase, PsychInfo, and Maternity and Infant Care databases on 16 April 2023. All articles retrieved were meticulously screened for eligibility by two independent reviewers with any disagreements resolved through discussion. The quality of the included articles was evaluated using the Mixed Methods Appraisal Tool. Studies were screened in Covidence and analysed in NVivo. The findings were organised and presented using Levesque’s framework of healthcare access.

**Results:**

Out of 558 identified papers, 11 studies comprising a total of 472 participants met the eligibility criteria. The review highlighted provider-side barriers such as shortage of information, unmet cultural needs, long waiting times, low engagement of women in care, discrimination, and lack of continuity of care. User-side barriers identified include communication issues, difficulty navigating the health system, and lack of trustful relationships with healthcare providers. In contrast, the review pinpointed provider-side facilitators including positive staff attitudes, service availability, and the proximity of facilities to residential homes, while user-side facilitators such as cultural assimilation and feeling valued by healthcare providers were noted.

**Conclusions:**

This review identified barriers and facilitators of access to maternity care for African-born women living in Australia. Empirical evidence that would inform potential changes to policy and practice to address African-born women’s unique health needs was highlighted. Designing and implementing a culturally safe service delivery model could remove the identified access barriers and improve African-born women’s engagement in maternity care. Moreover, reinforcing factors associated with positive healthcare experiences is essential for improving maternity care access for this priority population.

**Systematic review registration:**

PROSPERO CRD42023405458.

**Supplementary Information:**

The online version contains supplementary material available at 10.1186/s13643-024-02628-8.

## Background

Migration to high-income countries from low-middle-income countries has increased in recent years. According to the 2020 International Organization for Migration (IOM) report, about 3.6% of the world’s population has been living outside of their countries of birth [1]. The number of people living outside of their countries of origin had increased from 244 million in 2015 [2] to 281 million in 2020, a 15.2% increase over 5 years [1]. People often move to and live in another country as migrants, refugees, or asylum seekers, mainly when conditions in their birth country are inconducive to living a healthy and prosperous life [3].

According to the IOM definitions, an asylum seeker is someone who has sought international protection in another country due to serious human rights violations in their country of origin and is awaiting a final decision on their asylum claim [4]. A refugee is a person who has fled their birth country and lives in another country because of fear of persecution because of their race, religion, nationality, membership of a particular social group or political opinions [4]. Although there is no universally accepted definition for migrant, the United Nations High Commissioner for Refugees (UNHCR) considers it as an umbrella term covering people who willingly change their countries of origin for work (e.g. skilled migrants), temporary entrants (e.g. students), join a family (e.g. partner), or to lead a prosperous life [5]. A migrant can be classified either as a ‘first-generation immigrant’, a person who was born in a country other than the host country or a ‘second-generation immigrant’, a person who was born in a country of residence but to foreign-born parents [6].

Migration to Australia from countries across the world has increased over the last decade, making Australia one of the top 10 destination countries globally [7]. This is partly attributed to the country’s recent changes to migration policy, which has focused on attracting skilled migrants to fill skill shortages [8]. Australia is also a country of choice for many African migrants and refugees. Evidence has shown that in 2020, more than 400,000 people of African origin lived in Australia, representing 1.6% of the Australian population. About a third of African people living in Australia are women of childbearing age [9].

Addressing the health and well-being of migrants and refugees was recognised as an essential step toward achieving the Sustainable Development Goals (SDGs) [10]. However, access to healthcare can be challenging for migrant and refugee women residing in Australia, especially for those who are from non-English speaking countries [11]. Furthermore, most migrant and refugee African-born women could not easily adjust themselves to the health system of the destination countries, and thus often attend traditional practitioners [12].

The health and well-being of migrant and refugee women could be affected by exposure to health risks in their country of origin, during the transit, and in destination countries [13]. Due to these exposures and inequality in access to maternity care in the host countries [14, 15], migrant and refugee women are more likely to experience higher adverse perinatal outcomes when compared to women born in the host country [15]. Similarly, in Australia, where the government’s commitment to realise equitable health care is strong, adverse perinatal outcomes remain significantly higher among women with migrant or refugee backgrounds than women born in Australia [16, 17]. For example, perinatal mortality is significantly higher among migrant Eritrean women than Australia-born women (24.3 per 1000 births vs 9.8 per 1000 births) [18]. This might be attributed partly to the access barriers, health inequalities, or discrimination experienced by migrant women during healthcare service episodes [19].

With the rapid increase of migration from African countries to Australia, investigating the barriers and facilitators to maternity care access for African-born women is an essential step to realise equitable maternal health care. This is in line with the national strategic directions for Australian maternity services, which recognise the delivery of culturally safe, women-centered, and evidence-based models of care for women from culturally and linguistically diverse backgrounds [20].

Although a few studies have been conducted on the maternal health and well-being of migrant and refugee women in Australia, they do not reflect the unique needs of African-born women, as migrants and refugees are not a homogenous population [18]. The unique health, cultural, social, and psychological needs of this growing community need to be integrated into national health strategies [9]. Potential changes to health policy and practice are effective when supported with reliable and current evidence. Systematic reviews and meta-analyses are the best methods to supply accurate evidence for informed decisions [21]. However, there is a lack of systematically synthesised evidence on the barriers and facilitators of access to maternity care for African-born women living in Australia. This meta-synthesis aimed to synthesise current qualitative evidence on barriers and facilitators of access to maternity care among African-born women living in Australia.

## Methods

### Design

Relevant qualitative and mixed methods studies that have reported on barriers and/or facilitators to access maternity care among African-born women living in Australia were systematically searched. An a-priori analytic method was applied to present the findings derived from empirical studies using Levesque et al.’s conceptual framework of healthcare access [22]. A protocol for this review has been registered with the International Prospective Register of Systematic Reviews (PROSPERO) (registration number: CRD42023405458).

### Inclusion and exclusion criteria

The following eligibility criteria were used to identify relevant articles for the review. Peer-reviewed articles published in academic journals in the English language and reported on the barriers and/or facilitators of access to maternity care for African-born women living in Australia were included. Maternity care refers to the care provided to women during pregnancy, childbirth, and the postnatal period [23] and may occur in public or private health facilities. We included qualitative and mixed-methods studies, but we extracted the qualitative data reported in these studies. Two studies reported on women from some Asian and African countries, but only data specific to and quotes provided by African-born women were extracted.

Studies that reported on migrant and/or refugee women living in Australia but not of African origin were excluded. Access barriers to non-African migrant women might not be the same as access barriers to African-born women living in Australia. We excluded purely quantitative studies, commentaries, protocols, conference abstracts, and anonymous reports.

### Search strategy

Five online databases, including MEDLINE, CINAHL, Embase, PsychInfo, and Maternity and Infant Care (MIC), were searched on 16 April 2023 from the dates of inception without any time limits. These databases index pregnancy-related research papers. The search strategy and keywords were developed in consultation with an experienced faculty librarian. The study used the PICOS (Population, Interest, Comparison, Outcomes, and Study types) framework. This modified PICO shows higher sensitivity than SPIDER (Sample, Phenomenon of Interest, Design, Evaluation, and Research type) and greater specificity than PICO, thus is recommended in systematic reviews of qualitative literature [24]. It has been previously applied in a similar study [25]. Participant (P) refers to African-born women living in Australia, while the phenomena of Interest (I) is access to maternity care. Comparisons (C) are Australian-born women (this is implicit because all participants were African-born women). The Outcomes (O) are barriers and facilitators of access to maternity care, while the Study types (S) are qualitative and mixed methods studies.

A comprehensive search strategy and keywords were developed, and a line-by-line search method was conducted in candidate databases to locate suitable articles. Then, the search history options were combined using the Boolean operators (AND or OR) to make a final set of results. Search strategies and keywords for all databases via the EBSCOhost platform (Ovid for MIC) are found in a supplementary file (see Additional file 1). We conducted a pilot search and validated the candidate databases and keyterms because, pilot search is recommended as it improves the efficiency of the review [26]. Once searching was finalised, the bibliographic lists of the eligible papers were checked for relevant studies. The updated Preferred Reporting Items for Systematic Reviews and Meta-Analyses (PRISMA) flow diagram [27] was used to illustrate the selection process and the PRISMA checklist was used to report the reviews (see Additional file 2).

### Screening for articles

Articles were searched from the online databases by one reviewer (AGB) and saved to the EndNote library. Papers were imported into Covidence, duplicates were removed, and articles were shared with the second reviewer (VV) for the title and abstract screening, full-text review, and data extraction. The two reviewers conducted a comprehensive independent screening guided by the eligibility criteria. Disagreements were solved through discussion between the two reviewers and the articles were passed on to a third reviewer for a final decision (LS). Finally, the full texts of all articles that met the inclusion criteria were retained for meta-synthesis.

### Data extraction

Data were extracted from the full text of retained articles in Covidence using an adapted Joanna Briggs Institute (JBI) data abstraction format [28] (see Additional file 3). Study characteristics, including the authors names, publication year, data collection period, and the Australian state or territory in which the study was conducted, were extracted. Specific study details, such as the study design, study population, sample size, sampling procedure, and data collection methods were extracted from the included studies. We also extracted barriers and facilitators for maternity care access reported by African-born women.

### Quality appraisal

The quality of the papers was assessed using the updated Mixed Method Appraisal Tool (MMAT) [29]. We preferred to use the MMAT because it is suitable for assessing the quality of qualitative and mixed-methods studies. This tool has been validated and gained a moderate to perfect interrater reliability score [30]. The tool has been used for quality assessment in similar previous reviews [31, 32]. Two authors (AGB and VV) independently appraised the quality of all included papers. Using the tool, we determined the quality of the qualitative studies by examining (i) the appropriateness of the qualitative approach, (ii) whether the data collection methods were adequate, (iii) whether the findings were adequately derived from the data, (iv) whether the interpretation of results was sufficiently substantiated by data, and (v) whether there was coherence between qualitative data sources, collection, analysis, and interpretation. The quality of mixed-methods studies was determined by examining (i) whether there was an adequate rationale for using the methods, (ii) whether different components of the study were effectively integrated, (iii) whether the outputs of the integration of qualitative and quantitative components were adequately interpreted, (iv) whether divergences and inconsistencies between quantitative and qualitative results were adequately addressed, and (v) whether different components of the study adhered to the quality criteria of each method involved [29]. This tool has five quality assessment criteria, and the quality of each study is determined by summing up the number of all criteria met. The quality value ranges from 0% (no criterion met) to 100% (all criteria met) [33]. To capture all barriers and facilitators of access to maternity care for African-born women, all studies that meet the inclusion criteria were included, irrespective of their quality scores. However, the quality of each study was characterised and presented (see Additional file 4).

### Data synthesis

The current review was informed by Levesque and colleagues’ framework [22]. This conceptual framework offers a multidimensional view of healthcare access with five domains to assess the accessibility of services from the providers’ perspective including (1) approachability; (2) acceptability; (3) availability and accommodation; (4) affordability; and (5) appropriateness. This framework also incorporates five corresponding abilities that are appropriate to assess the accessibility of service from the users’ perspective including (1) the ability to perceive; (2) the ability to seek; (3) the ability to reach; (4) the ability to pay; and (5) the ability to engage [23]. This framework is suitable to investigate the accessibility of services both from users’ and providers’ perspectives, not just the failures of the health system [22]. Furthermore, unlike other available frameworks, Levesque and colleagues offer dimensions such as approachability and appropriateness of the services, which are specifically relevant among socially disadvantaged and migrant women [34, 35]. Although this framework has some challenges, such as the difficulty of categorising certain data into a specific dimension [36], it is highly regarded as appropriate and has been widely used in the literature [22, 34–37] to organise and present barriers and facilitators to healthcare access.

**Fig. 1:**
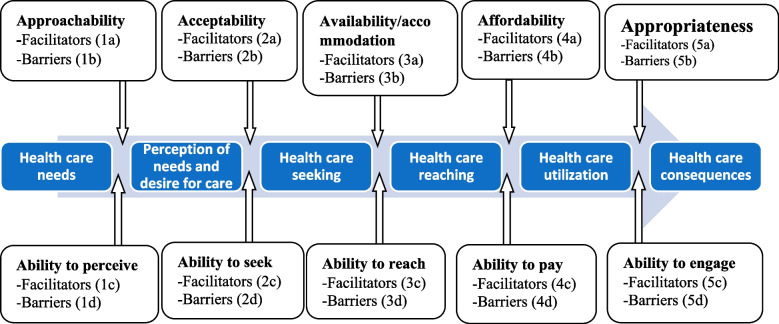
Adopted Levesque and colleagues’ conceptual framework for healthcare access

Guided by Levesque and colleagues’ conceptual framework of healthcare access [22], we coded the data line-by-line and generated various codes and sub-themes. These were then grouped into the broader domains within the framework. The data were coded into Levesque et al.’s dimensions by the first author and checked by the other authors for precision. Finally, we selected illustrative quotes from the extracts to reflect the broader meaning of the domain. To remove ambiguity in the classification of findings, we named provider-side facilitator (a), provider-side barriers (b), user-side facilitators (c), and user-side barriers (d), preceded by the corresponding number of domains. For example, 2a represents facilitators in the acceptability domain (Fig. 1). Data management, coding, and presentation were performed with Nvivo Version 20 software.

### Assessment of confidence in the review findings

Once we finalised the qualitative evidence synthesis, we assessed the level of confidence in the findings using the Grading of Recommendations Assessment, Development and Evaluation-Confidence in the Evidence from Reviews of Qualitative research (GRADE-CERQual) [38–42]. Initially, we assessed each discrete finding reported in the qualitative evidence synthesis based on the methodological limitations of the studies contributing to the finding, the coherence of the finding, the adequacy of data contributing to the finding, and the relevance of the contributory studies to the review question. Then we made an overall judgement and categorised each finding as having High, Moderate, Low or Very Low confidence. We set an initial assumption of ‘high confidence’ in all findings and downgraded the levels based on the criteria described in the protocol. The GRADE-CERQual assessments were performed by two reviewers independently and a final judgement was made based on discussions and consensus [40].

## Results

**Fig. 2:**
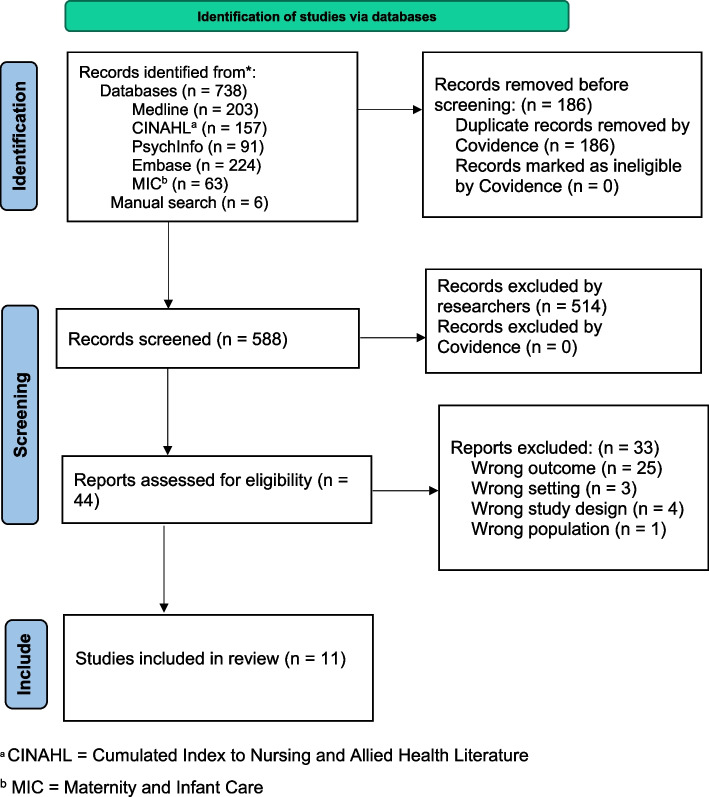
PRISMA flow diagram indicating searches of databases, article screening and selection

After comprehensive screening, eleven studies that met the eligibility criteria were retained for the qualitative evidence synthesis [43–53]. These studies were published between 2003 and 2022. Ten studies employed only qualitative methods [43–45, 47–53], typically using interviews and focus group discussions for data collection. One study used a mixed methods approach [46]. Participants of nine studies included only African-born women [43–47, 49–51, 53], while two studies presented data from women of African and Asian backgrounds [48, 52]. Responses specific to African-born women were extracted from these studies. The included studies were conducted in five Australian states, with most from Victoria [43–45, 49] and Queensland [46, 51, 53] (Table 1). Seven studies [43–45, 47, 48, 50, 52] met all quality criteria, while four studies [46, 49, 51, 53] met four out of the five criteria. Article screening and the selection process are illustrated in the PRISMA flow diagram [54] (Fig. 2).

**Table 1:** Characteristics of the studies included in the qualitative evidence synthesis

Studies	Aims of the primary studies	State	Study type	Participants’ country of origin	Number of respondents	Sampling technique	Data collection methods	Data collection period	Analysis method	Quality score
Benza and Liamputtong 2017 [43]	To discuss the meanings and experiences of motherhood from the perspectives of Zimbabwean migrant women	VIC	Qualitative	Zimbabwe	15	Theoretical sampling method	In-depth interviews with photo and drawing elicitation methods	Not stated	Thematic analysis	5
Carolan and Cassar 2007 [44]	To explore factors that facilitated or impeded the uptake of antenatal care among African refugee women	VIC	Qualitative	Sudan, Ethiopia, Somali, Eritrea, Kenya	10	Voluntary	Interview and observation	Not stated	Thematic analysis	5
Carolan and Cassar 2010 [45]	To explore the experiences and concerns of an African-born sample of pregnant women receiving antenatal care	VIC	Qualitative	Ethiopia, Sudan, Eritrea, Somalia, Kenya	18	Purposive	In-depth interview	2006–2007	Thematic analysis	5
Correa-Velez and Ryan 2012 [46]	To determine the key elements that characterise the best practice model of maternity care for women from refugee backgrounds and developing and implementing such a model at the Mater Mothers’ Hospital	QLD	Mixed methods	Sudan, Burundi, Ethiopia, the Democratic Republic of Congo, and Somalia	23	Purposive	Interview and questionnaire	April–May 2008	Thematic analysis	4
Due et al 2022 [47]	To understand the relationship between psychological wellbeing and perinatal care amongst African refugees and identify areas for improved perinatal healthcare services to ensure positive wellbeing outcomes	SA	Qualitative	Sierra Leone, Liberia, The Democratic Republic of Congo, Somalia, Ethiopia, Burundi, Ghana, and Nigeria	19	Purposive	Interview	July 2019 and December 2020	Thematic analysis	5
Hawkey et al 2022 [48]	To identify migrant and refugee women’s preferences for Sexual and Reproductive Health information and service delivery	NSW	Qualitative	Somalia, South-Sudan, and Sudan	86 (61 from interviews and 25 from Focus Group Discussions)^a^	Voluntary	Interview and Focus Group Discussion	July 2014 and November 2015	Thematic analysis	5
Manderson and Allotey 2003 [49]	To investigate cultural understandings of reproductive rights and reproductive health factors that hindered or enhanced the use of reproductive health services and to identify indicators of the general wellbeing of migrant and refugee women	VIC	Qualitative	Sudan, Somalia, Eritrea, and Ethiopia	255 surveys, 150 from interviews and 105 from Focus Group Discussions ^b^	–	Case studies^c^	1999 to 2001	Descript-ive analysis	4
Mohale et al 2017 [50]	To examine the maternity experiences of Sub-Saharan African women who had given birth in both Sub-Saharan Africa and in Australia	SA	Qualitative	Liberia, South-Africa, Uganda, Malawi, Burundi, South Sudan, and Sudan	14	Purposive and snowball	Interview	Not stated	Thematic analysis	5
Murray et al 2010 [51]	To uncover first-person descriptions of the birth experiences of African refugee women in Brisbane, Australia, and to explore the common themes that emerged from their experiences	QLD	Qualitative	Sudan, Liberia, Somalia, and Ethiopia	10	Purposive and snowball	Interview	Not stated	Thematic analysis	4
Owens et al 2016 [52]	To explore the perceptions of care experienced by refugees and migrant women of culturally and linguistically diverse backgrounds who had participated in a community-based antenatal programme specialising in maternity care of multi-cultural women	WA	Qualitative	Sudan^d^	12 (1 Sudan)	Purposive	Interview	June to November 2014	Thematic analysis	5
Tyler et al 2014 [53]	To highlight and compare immigrant Sudanese women’s infant feeding choices and patterns before and after moving to a regional city in Queensland, Australia	QLD	Qualitative	Sudan	10	Voluntary	Interview	Not stated	Thematic analysis	4

^a^This study included migrant women from Afghanistan, India, Iraq, Somalia, South Sudan, Sri Lanka, Sudan, and Latina. Participants were 84 interviewees (61 in Sydney and 23 in Vancouver) and 16 focus group discussion participants, giving a total of 85 participants (25 participants in Sydney and 60 in Vancouver). The results did not identify whether the respondents were in Canada or Australia, but we extracted the responses specific to African-born women

^b^This paper presented the case studies drawn from prior research, including surveys, interviews, and discussions of focus groups

^c^The sample size for the focus group discussion was not mentioned

^d^This study included one African woman (Sudan) and 11 Asian Women (Burma, Thai, Iran, Indonesia, Pakistan, and Vietnam). Data specific to the one African woman participant were extracted

### Qualitative evidence synthesis

The qualitative findings reported in the included studies are presented below under five Levesque et al.’s conceptual framework of healthcare access domains [22], and details of the themes are presented in Table 2. We also presented the barriers and facilitators of access to maternity care excerpted from all studies in Table 3.

**Table 2:** Barriers and facilitators identified from the included studies

Facilitators	Studies	Barriers	Studies
1) Approachability and ability to perceive			
1a) Provider-side facilitators		1b) Provider-side barriers	
➢ The provision of maternal health education in some hospitals	[46]	✓ Weak maternal health information dissemination	[43, 45]
		✓ Provision of information only in written form	[50]
1c) User-side facilitators		1d) User-side barriers	
➢ Women have a good interest in receiving maternal health information	[45]	✓ Lack of awareness about the available maternity care options	[50, 53]
		✓ Lack of familiarity with some interventions	[45, 51]
		✓ Misunderstanding why the services are given for	[49]
		✓ Not knowing the health system, the treatment standards, and the available antenatal education	[51]
		✓ Lack of knowledge of the locations of maternity services	[53]
		✓ Considering birth is a natural process, medical intervention is not required	[45, 51]
		✓ Worrying about the fetus accepting some procedures	[45]
		✓ Lack of trust in service providers	[48, 49]
		✓ Negative beliefs about medical interventions	[49]
		✓ Perceiving that health education is not important	[50]
		✓ Feeling alone and feeling different in the hospital environment	[44, 51]
		✓ Obstetricians are perceived as not concerned for women’s wellbeing	[51]
2. Acceptability and ability to seek			
2a) Provider-side facilitators		2b) Provider-side barriers	
➢ Positive staff attitude and respectful staff	[44, 50]	✓ Lack of respectful service delivery and discriminated	[43]
➢ Staff respect women’s culture	[45, 47]	✓ Care was not responsive to women’s cultural needs	[45, 47]
➢ Allowing families to present and support birthing mothers	[50]	✓ Care providers rush during the consultation	[48]
➢ Being asked about wellbeing by staff	[51]	✓ Restriction of movement during labour	[50]
➢ Midwives are kind	[51]	✓ Midwives posed inappropriate questions during service delivery	[51]
➢ Having access to a bicultural social worker	[47]	✓ Some staff are angry at women during service delivery	[51]
		✓ Unmet need for female doctors’ preference	[51]
		✓ Not involving family during labour	[46, 48]
		✓ Being labelled and receiving racially stereotypical comments in the hospital environment	[43]
2c) User-side facilitators		2d) User-side barriers	
➢ Women feel that service providers understand them	[52]	✓ Difficulties understanding different approaches to maternity care	[45]
➢ Feeling welcome and valued by the providers	[44]	✓ Home birth experience in the home country	[45]
➢ Cultural assimilation helped women to accept the Australian health system	[45]	✓ Being distressed when technology was used	[46]
		✓ Prior negative maternity care experiences	[47]
		✓ Religious restriction	[46, 49]
3) Availability and accommodation, and ability to reach			
3a) Provider-side facilitators		3b) Provider-side barriers	
➢ Availability of multiple forms of pain relief	[50]	✓ Postnatal follow-up is not available	[43]
➢ Availability of antenatal classes during visits	[51]	✓ Long waiting time	[46, 50]
➢ Multiple services are provided under one roof	[52]		
➢ The proximity of health facilities to home	[44, 52]		
3c) User-side Facilitators		3d) User-side Barriers	
➢ We could not associate any finding to this theme		✓ Difficult accessing public transport	[46]
		✓ Difficulty navigating the health system and the hospital environment	[51]
4) Affordability and ability to pay			
4a) Provider-side facilitators		4b) Provider-side barriers	
➢ We could not associate any finding to this theme		✓ We could not associate any finding to this theme	
4c) User-side facilitators		4d) User-side barriers	
➢ We could not associate any finding to this theme		✓ Financial constraints and problems related to Medicare	[45]
		✓ Loss of job/income due to childcare commitment	[46]
		✓ Resettlement is a priority over their healthcare	[45]
5) Appropriateness and ability to engage			
5a) Provider-side facilitators		5b) Provider-side barriers	
➢ Being accepted by the healthcare provider	[45, 52]	✓ Lack of continuity of care	[47, 51]
➢ Care is given by a midwife throughout	[46, 50]	✓ Suboptimal quality of maternity care	[47]
➢ Use of health technology for maternity care	[50, 51]	✓ Midwives lack experience in providing care for women with circumcision	[46, 51]
➢ Healthcare providers are supportive	[52]	✓ Lack of consent for the presence of students in labour ward	[47]
➢ Communicative approach to midwifery care	[47]	✓ Problems related to interpreter services such as gender, late arrival, confidentiality, and lack of awareness about the service	[46, 50, 51]
➢ Presence of an appropriate interpreter	[45, 46]	✓ Women’s requests being ignored by service providers	[47]
➢ Positive experiences in relation to consent	[47]	✓ Women not being recognised for their prior pregnancy experiences	[47, 51]
5c) User-side facilitators		5d) User-side barriers	
➢ Meeting other African fellow women at the facility	[44]	✓ Lack of awareness about the availability of continuity of care	[50]
➢ Learning English created a sense of empowerment	[51]	✓ Language/communication barriers	[45, 50, 51, 53]
		✓ Not explaining to women what services are given and why for	[49]
		✓ Being not listened to or have no input/control over their experiences	[47]

**Table 3:** Barriers and facilitators of access to maternity care among African-born women living in Australia

**Barriers**	Studies
➢ Weak maternal health information dissemination	[43, 45]
➢ Provision of information only in written form	[50]
➢ Lack of awareness about the available maternity care options	[50, 53]
➢ Lack of familiarity with some interventions	[45, 51]
➢ Misunderstanding why the services are for	[49]
➢ Not knowing the health system and the available antenatal opportunities	[51]
➢ Lack of knowledge of the locations of maternity services	[53]
➢ Considering birth is a natural process, medical intervention is not required	[45, 51]
➢ Worrying about the fetus accepting some procedures	[45]
➢ Lack of trust in service providers	[48, 49]
➢ Negative beliefs about medical interventions	[49]
➢ Perceiving that health education is not important	[50]
➢ Feeling alone and feeling different in the hospital environment	[44, 51]
➢ Obstetricians are perceived as not concerned for women’s wellbeing	[51]
➢ Lack of respectful service delivery and discriminated	[43]
➢ Care was not responsive to women’s cultural needs	[45, 47]
➢ Care providers rush during the consultation	[48]
➢ Restriction of movement during labour	[50]
➢ Midwives ask inappropriate questions the women	[51]
➢ Some staff are angry at women during service delivery	[51]
➢ Unmet need for female doctors’ preference	[51]
➢ Not involving family during labour	[46, 48]
➢ Being labelled and receiving racially stereotypical comments	[43]
➢ Difficulties understanding different approaches to maternity care	[45]
➢ Home birth experience in the home country	[45]
➢ Being distressed when technology was used	[46]
➢ Prior negative maternity care experiences	[47]
➢ Religious restriction	[46, 49]
➢ Postnatal follow-up is not available	[43]
➢ Long waiting time	[46, 50]
➢ Difficult accessing public transport	[46]
➢ Difficulty navigating the health system and the hospital environment	[51]
➢ Financial constraints and problems related to Medicare	[45]
➢ Loss of job/income due to childcare commitment	[46]
➢ Resettlement is a priority over their healthcare	[45]
➢ Lack of continuity of care	[47, 51]
➢ The quality of maternity care was perceived as suboptimal	[47]
➢ Midwives lack experience in providing care for women with circumcision	[46, 51]
➢ Lack of consent for the presence of students in labour ward	[47]
➢ Problems related to interpreter services such as gender, late arrival, confidentiality, and lack of awareness about the service	[46, 50, 51]
➢ Women’s requests being ignored by service providers	[47]
➢ Women not being recognised for their prior pregnancy experiences	[47, 51]
➢ Lack of awareness about the availability of continuity of care	[50]
➢ Language/communication barriers	[45, 50, 51, 53]
➢ Not explaining to women what services are given and why for	[49]
➢ Being not listened to or have no input/control over their experiences	[47]
**Facilitators**	Studies
➢ The provision of maternal health education in some hospitals	[46]
➢ Women have a good interest in receiving maternal health information	[45]
➢ Positive staff attitude and respectful staff	[44, 50]
➢ Staff respect women’s culture	[45, 47]
➢ Allowing families to present and support birthing mothers	[50]
➢ Being asked about well-being by staff	[51]
➢ Midwives are kind	[51]
➢ Having access to a bicultural social worker	[47]
➢ Women feel that service providers understand them	[52]
➢ Feeling welcome and valued by the providers	[44]
➢ Cultural assimilation helped women to accept the Australian health system	[45]
➢ The availability of multiple forms of pain relief	[50]
➢ The availability of antenatal classes during visits	[51]
➢ Multiple services are provided under one roof	[52]
➢ The proximity of health facilities to the home	[44, 52]
➢ Being accepted by the healthcare provider	[45, 52]
➢ Care is given by a midwife throughout	[46, 50]
➢ Use of health technology for maternity care	[50, 51]
➢ Healthcare providers are supportive	[52]
➢ Communicative approach to midwifery care	[47]
➢ Presence of an appropriate interpreter	[45, 46]
➢ Positive experiences in relation to consent	[47]
➢ Meeting other African fellow women at the facility	[44]
➢ Learning English created a sense of empowerment	[51]

#### Perception of needs and desire for care

##### Approachability

Approachability refers to the availability of adequate information about existing services and how people identify and reach for care [22]. The provision of maternal health education at the hospitals was considered a provider-side facilitator (1a) that supported African-born women to access maternity care [46]. However, African-born women reported several provider-side barriers (1b) for accessing maternity care in Australia. Most women claimed that a lack of maternal health information [43, 45] limited their understanding of the purpose of the services [48]. Due to a lack of knowledge about the available maternal health options [50] and a lack of knowledge about where to obtain the services [53], some women missed important maternity care appointments available at the early stage of pregnancy. A woman presented her concern: ‘Because it was my first time having a child here, and I don’t know exactly the places I have to go like hospitals. … I stayed at home until I was 7 months pregnant, I hadn’t been even to first check-up’ [50, p: 301].

##### Ability to perceive

The ability to perceive is related to health literacy, knowledge, and beliefs about health and sickness [22]. It was reported that some women are interested in receiving information about maternal health, which we identified as the only user-side facilitator (1c) [45]. However, several user-side barriers (1d) were reported to affect women’s ability to perceive. Most women perceived childbirth as a natural process with medical assistance, including analgesia for labour pain [45, 51] and medical procedures during pregnancy [45, 49] being perceived as not necessary. The use of medical technology during labour was perceived as distressing for some women [46]. Therefore, some women preferred to labour at home for as long as possible, believing this would prevent birth interventions, including caesarean section [51]. For example, a woman reported rejecting medication for labour induction because she wanted only a natural birth [45, 48, 49], feeling alone and feeling different from others in the hospital environment [44, 51], and women’s belief in complementary (natural) therapies over modern medicine [51] were reported to hamper care seeking behaviour of African-born women. Here is a perception of a woman who avoided analgesia in the belief that it prolongs labour by cooling the pain: ‘… when you are on labour, then, um, instead you are given hot water, tea, hot tea, dry tea. … for the tablets, … it will cool the pain and still the baby will remain in me. So, I just want the pain should (escalate) so that it [baby] comes out’ [51, p: 467].

#### Health care seeking

##### Acceptability

Acceptability relates to how well healthcare services address an individual’s cultural and social needs for them to seek, access, and accept care [22]. Several provider-side factors, such as the welcoming and positive attitudes of midwives [44, 50], being understood by midwives and doctors [44, 52], and having access to bicultural social workers [47] facilitated (2a) African-born women to accept and access the services. Some women considered midwives as very kind [51] and were respectful to women’s culture during service provision [47], which helped to build trustful relationships [45]. African-born women appreciated being asked about their well-being during inpatient stays [51] and families being allowed to be present and provide support during childbirth [50]. For example, a respondent narrated: ‘…so, she tries to meet your needs in the way that you feel comfortable… so I think for her, she treats everybody the way they’re meant to be treated, because she doesn’t treat everybody the same. She kind of – she understands what people’s individual needs are…’ [47, p: 4].

Conversely, a range of provider-side barriers (2b) were reported to affect the acceptability of the services by African-born women. Discrimination [43], unmet needs for maternity care [47], and insufficient time for consultations [48] were reported to affect women’s access to maternity services. Furthermore, women’s care-seeking behaviour was diminished if their preference for female doctors was not met and when staff were angry and failed to respond to their requests [51]. For example, a woman narrated her concerns about service providers: ‘I don’t blame them. I know they are busy, but … You feel you need someone, you know, even if you want something and you press for the nurse, and they don’t come. You feel like … maybe they don’t want you; they don’t want to help you’ [51, p: 465].

##### Ability to seek care

The ability to seek care is related to personal autonomy, knowledge about the available options, and the freedom to obtain care [22]. A range of user-side facilitators (2c) were reported to improve African-born women’s ability to seek maternity care. Cultural assimilation helped some women develop the confidence to accept the Australian hospital system [45]. Some women felt welcomed and valued by service providers, which improved their ability to seek maternity care [44].

However, many user-side barriers (2d) were reported across studies to affect women’s ability to seek maternity care. Women’s religion was reported as a factor that affected the acceptability of maternity care [46, 49]. For example, a respondent narrated that she would rather die a good Muslim woman than be ‘contaminated’ by having a ‘pork injection’ (routine Heparin injection), which was believed to be a non-halal product [49, 45]. Being asked for private and personal information, such as whether women had been circumcised, was also considered culturally unsafe [47]. Some women preferred not to visit maternity services as they were being labelled and received racially stereotypical comments in hospitals [43]. As described by one respondent: ‘I think it’s hard and people already have these thoughts about you as you walk through the door, like as they see this African woman, they think oh she’s here to cause trouble. I even heard another midwife saying, ‘She should be happy that she’s in Australia” [43, p: 76].

Furthermore, a lack of social support, such as limited family involvement during labour [46, 48] was reported to affect African-born women’s ability to seek care. Prior negative maternity care experiences [47] and a perception of being ignored in hospitals [53] challenged some women's care-seeking behaviour. Women reported being unhappy with attending the service facilities when their culture was not respected, and the hospital food did not meet their religious requirements [46]. A respondent narrated*:* ‘I feel the staff have little understanding about my culture and … the importance of family during labour. My family was sent home... nobody informed me as to what happened … my family are not happy about it. Food was inappropriate…’ [46, p: 19].

#### Health care reaching

##### Availability and accommodation

Availability and accommodation are defined as the physical existence of health resources within a geographical location with sufficient capacity to deliver care promptly [22]. Various provider-side facilitators (3a) that improved African-born women’s access to maternity services were related to this domain. Many women across the studies were positive about the availability of services in Australian hospitals [50–52]. Maternity services were close to women’s residential homes [44, 52], making accessing care easy. African-born women were happy to visit a facility where they could get multiple services at one point of care [52].

Nevertheless, women reported a few provider-side barriers (3b) that affected their access to maternity care. Despite the satisfactory midwifery care in hospitals, some women described a lack of postnatal follow-up services [43]. Other women reported being frustrated with visiting maternity services as the hospital waiting time was too long [46, 50]. One woman complained: *‘Long waiting with an empty tummy or will be booked for morning but sometimes it takes 3–5 h waiting…’* [46, p: 16].

##### Ability to reach

The ability to reach healthcare is defined as the personal mobility and availability of transportation that would enable the users to physically reach the service [22]. Although we could not associate any user-side facilitator (3c) to this domain, a few user-side barriers (3d) were reported to impede African-born women’s ability to reach and use the available maternity services. Although all maternal health services were reportedly available [52], difficulties navigating the health system [51] diminished women’s ability to reach the services they needed. Furthermore, difficulty accessing public transport to get to a maternity service was reported as a barrier for some women [46]. For example, a respondent mentioned: ‘…Transport was an issue for me. Sometimes I get to the hospital for my appointment late’ [46, p: 16].

#### Health care utilization

##### Affordability

Affordability relates to the economic capacity of people to spend resources and time to use appropriate services. This includes direct and indirect costs such as the inability to work and subsequent loss of income to attend to healthcare [22]. None of the reported barriers and facilitators could be associated with this domain (4a and 4b).

##### Ability to pay for service

The ability to pay relates to the capacity to generate income to cover the cost of health services without catastrophic expenditure [22]. We could not identify any facilitators (4c) to this theme; however, we associated a few user-side barriers (4d). Some women gave priority to resettlement over their healthcare [45] thus, they missed some important maternity care appointments. A lack of sufficient money and unemployment due to childcare commitments affected women’s ability to pay for services [45, 46]. A respondent reported her concern about her ability to pay: ‘… I had no family, … there was a problem with Medicare (waiting period) and we had to pay $35 to see the doctor we have no money…’ [45, p: 195].

#### Health care consequences

##### Appropriateness

Appropriateness refers to promptly providing quality health services to qualified healthcare providers, improving user engagement [22]. Many provider-side facilitators (5a) motivated African-born women to be engaged in maternity care in Australia. Some African-born women valued the continuity of midwifery care, allowing them to be cared for by the same midwife throughout their perinatal period [46, 50]. The supportive care women received from service providers [45, 52] motivated them to revisit the facility for maternity care. Despite being unfamiliar with some procedures, such as an ultrasound, some African-born women related this to better quality of care and were willing to use maternity service technology [50, 51]. For example, a participant who was told she was having a baby girl during an ultrasound examination and then gave birth to a baby girl suggested that this created trust in the services she received and motivated her to revisit [51, p: 464].

Several provider-side barriers (5b) were reported across the studies. A few women complained about the lack of continuity of care [51], as different midwives attended to them every visit [47]. Participants stated they were frustrated introducing themselves to new staff at each service appointment, which they described as ‘really exhausting and challenging’ [51, 50] as a barrier to access. One study participant indicated, ‘… somebody told me it is your choice; you can have the same midwife until you give birth. But in my case, I didn’t have…. I didn’t know about that’ [50, 47]. For example, a woman who was not happy with the quality of her maternity care perceived that she was treated as a ‘guinea pig’ [47, 47, 51]. One study participant explained: ‘She looked really panicked when she tried to, um, to deliver the baby, and she didn’t know what to do, but I had my niece to tell her quickly that she can cut…. I felt I was different because of the female circumcision I had and I wasn’t really sure. I felt so embarrassed the whole time that I was at the hospital’ [51, p: 466].

Most African-born women recounted instances of feeling that they were not listened to nor able to have input or control over their health care [47], which subsequently diminished their engagement in maternity care. Some service providers did not explain to women why the services were given [49]. Women in a study complained that their request for female midwives was ignored [47]. A participant described, ‘I did have a female, and then they switched without telling me. Then when I came in, it was a male. Then I said no, I don’t want to see a male as a midwife. I want a female but even up until now, when I go, I still see male’ [47], p: 12. Furthermore, not being recognised for a previous birthing experience [47, 51] and not being asked for consent [47] hampered women’s engagement in the services. Such experiences created fear among women to discuss their circumstances [44], creating a reluctance to ask questions [51] about the care they were receiving.

##### Ability to engage

The ability to engage in health care is related to the client’s involvement in decision-making about treatment, which motivates them to participate in care [22]. It was reported across the studies that some user-side facilitators (5c) improved women’s engagement in maternity care. Learning English created a sense of empowerment and improved women’s engagement in maternity care [51]. Some African-born women were interested in visiting health facilities to meet other African-born women to share information [44].

Many user-side barriers (5d) were reported across the studies that limited engagement with maternity services. Communication barriers were widely reported to hinder African-born women’s access to maternity care [46, 50]. Although some African-born women could speak English, they found it difficult to understand the staff who spoke too quickly and used slang words [45, 50, 51]. Women felt that the service providers did not understand the English they spoke [50]. A participant in a study described such communication difficulties*:* ‘Communication is a big problem with me because my English is not good. Every time I speak to them, they don’t understand me I think they don’t get my English very well’ [50, 46, 50, 51]. Some African-born women described that the late arrival of interpreters delayed their appointments [46]. Negative experiences related to the age and gender of the interpreters [46] and concerns about confidentiality [50] were reported as barriers to using the services. In some cases, women lacked awareness about the availability of interpreting services [51]. A participant explained problems regarding interpreter use: ‘Interpreters from Syria and Iraq can’t understand us and we can’t understand them. I prefer interpreter from Sudan [who speaks Sudanese Arabic]. … I was provided with a young girl interpreter… she was unfamiliar with women problems’ [46, 46

### Level of confidence in the findings: GRADE-CERQual

Eighteen discrete findings were subjected to GRADE-CERQual confidence assessments. Additional file 5 presents the detailed Evidence Profile and rationale for judgements in each of GRADE-CERQual’s four components. Overall, confidence in the findings of this qualitative evidence synthesis was either high (*n* = 8 findings) or moderate (*n* = 10 findings), providing reassurance for the applicability of the findings for informing policy and practice. Most of the downgrading in confidence related to the methodological limitations was affected by the quality of the studies contributing to the findings.

## Discussion

This review synthesised barriers and facilitators of access to maternity care in African-born women living in Australia guided by Levesque et al.’s conceptual framework [22]. We drew a comprehensive list of barriers and facilitators reported in the available literature and associated these with provider-side or user-side characteristics that collectively indicated the status of access to maternity care among African-born women living in Australia.

The current review identified several provider-side issues related to the approachability of the service. The routine health education provided for women in hospitals was a provider-side facilitator that improved access to maternity services. Health education helps women to make informed decisions about their care needs [55]. Conversely, a lack of relevant maternity information impedes women’s access to care [34]. Lack of information can also limit women’s knowledge about available care options [34, 56, 57], and awareness of the health system structure [58]. Informed maternity services are highly relevant for migrant women [57] but the importance of health information is often overlooked often due to inadequate consultation times [48]. Although information about maternity care may be available to the public, migrant women have less exposure to such information [59, 60]. This could be because of cultural and language barriers or low societal positioning of migrant women in a new country [60].

A range of user-side facilitators and barriers related to women’s ability to perceive maternity care were identified. African-born women have an interest in exploring further information about their maternity care. Women might need information to adapt to the new health system [61] and to make informed decisions during pregnancy [60]. Otherwise, they might choose to use complementary therapy and traditional approaches over attending facilities for maternity care [12]. A lack of information in an accessible language or format may hamper a trustful relationship affecting the quality of maternity care [60, 62]. Mistrust could also arise when the service conflicts with the cultural norms and preferences of migrant and refugee women [63].

Many provider-side facilitators enhanced African-born women's acceptability of maternity care in Australia. When healthcare providers show a positive attitude toward migrant women [60] and take their concerns seriously [64] women’s access to maternity services is improved [65]. A negative attitude, such as being unfriendly, disrespectful, and ignoring women’s concerns often leads to a loss of connection, and women tend to reject services [60]. Discrimination, which is an important source of disparity in access to maternity care, was also reported by women [15].

The review pinpointed a range of user-side facilitators and barriers regarding women’s ability to seek maternity care. Similar to the findings of a previous study [66], African-born women who were culturally assimilated have better access to maternity care. Although migrant women intend to keep their traditions and cultures in the new country [67], acculturation removes sociocultural barriers for women to explore and access the available services [68]. However, women’s cultural needs affected women’s ability to seek care. Our findings are not uncommon; migrant women value healthcare providers who understand and respect their cultural and religious needs [35].

Moreover, service providers need to be culturally competent and have an awareness of the cultural needs of migrant women [63]. Cultural and traditional preferences are highly relevant for migrant women to access maternity care but are often overlooked [57]. Although acculturation might help to integrate migrant women into the health system, this takes longer time for African-born women to fully integrate into the new country’s culture [66]. Most migrant women prefer female healthcare providers [34] because women are more open to speaking about their health needs to female healthcare providers [69].

Regarding the availability and accommodation of maternity services domain, the proximity of health facilities to women’s residential addresses improved attendance at maternity care [34, 57]. Nevertheless, the literature indicates that provider-side barriers such as inadequacy of postnatal follow-up [15] and long waiting times reduce the frequency of access to maternity care among migrant women [34].

Although we could not associate any user-side facilitators with the ability to reach maternity services, a few user-side barriers were reported Consistent with our review findings, difficulty navigating the health system in host countries for migrant women is commonly reported [34, 37, 63, 70]. This could be attributed to a lack of information about where to go for services or a lack of transportation [61]. To reduce the effect of transportation on access to maternity care, the World Health Organization recommends providing services at local clinics to avoid the need for long-distance travel to hospitals [70]. This could also relieve the financial constraints related to transportation costs that affect service access by migrant African-born women [60].

Many provider-side facilitators and barriers related to the appropriateness of maternity services were noted. Continuity of the midwifery care model has improved access to maternity care in African-born women [34, 35, 60]. Migrant women might prefer continuity of midwifery care because they often perceive that the consultation times they receive from clinicians are insufficient [35]. Furthermore, when different midwives attend to women at each visit, they are less likely to re-attend for appointments [71]. Continuity of care is essential, especially for African-born women who have experienced circumcisions because they perceive that midwives lack the necessary experience and training to provide need-based care. This could impact their engagement with maternity services [63, 72]. Circumcised women may be embarrassed to attend health care as they feel their vulva is ‘ugly’ [72]. Even those women who attended the care facilities preferred to undergo a caesarean section rather than vaginal birthing [63], indicating that discussing women’s needs and preferences is important.

Allowing women to control their own health needs is necessary to enhance their engagement in maternity care [60]. However, the current findings suggested that African-born women in Australia do not feel involved in care decision-making. Migrant women often lack appropriate information for making informed decisions [55], or healthcare decisions are often made by other family members such as husbands [63]. Therefore, empowering migrant women [22] by providing them with culturally appropriate information [34, 55, 61], can enhance informed decision-making. Recognising and discussing women’s previous birthing experiences [34, 56] can reduce women’s reluctance to disclose their circumstances (such as circumcision). This could also minimise women’s belief that midwives have more authority than themselves to make appropriate care decisions [65].

Effective communication is extremely important to women to discuss their health concerns with healthcare providers [60, 73]. However, migrant women often face significant language barriers in host countries [57, 60, 74]. This could impact their ability to make appointments [57], and their relationship with service providers [60, 75], limiting their access to maternity care [56, 76]. Poor communication could emerge from a lack of information in an accessible language [60, 77–80]. Information obtained from service providers often conflicts with cultural advice [60, 61, 80, 81], posing insecurity to women about which actions to take [60, 73, 81]. Even women who learned the English language still lack a vocabulary [60, 64, 75] to communicate effectively with service providers. A well-designed interpreter service is a good strategy to remove language barriers, but the quality of the service was found to be variable. A poor quality interpreter service often leads to misunderstanding of health messages [34, 60, 82] and may embarrass women during service encounters [60, 83]. Thus, interpreters must be used with caution especially when discussing intimate matters [83, 84]. Wherever possible, family members and friends might be used to interpret as clients often prefer this approach [85].

## Strength and limitations

The review has the following strengths. The review mapped domains of barriers and facilitators to access maternity care from both provider-side and user-side perspectives. The review findings are representative of the Australian context, with included studies conducted in five Australian states. Generally, barriers and facilitators to maternity care access were identified from articles with diverse study designs and presented using an appropriate  conceptual framework [22]. The limitations of the current review include the inclusion of articles published only in English. We did not include grey literature, including government reports; hence, we might have missed some potentially relevant studies indexed elsewhere or published in a language other than English.

## Conclusions

The current meta-synthesis found that both user-side and provider-side factors hindered or enabled African-born women’s access to maternity care. However, barriers to maternity care access were more frequently reported in the literature than the facilitators. The majority of the barriers were related to the acceptability and appropriateness of services [22]. This implies that cultural beliefs, religion, traditional customs, communication, and respectful provider-user relationships are highly pertinent in maternity care for migrant African-born women. This highlights the importance of designing culturally safe maternity service delivery models considering the unique needs of migrant African-born women. This review presented relevant findings to inform the development of appropriate interventions to enhance equitable maternity care access for African-born women living in Australia. The review provided robust evidence to inform maternal health policy and practice for African-born women living in Australia and beyond. Barriers encountered due to language barriers could be removed by the consistent use of interpreters and by ensuring communication materials are being prepared and delivered to women in a language they prefer. Cultural sensitivity training for maternal health service providers might be helpful to accommodate women’s cultural needs during service episodes. Future research is needed to assess whether the Australian health system is responsive to the unique health needs of migrant African-born women.

### Supplementary Information

Additional file 1: PRISMA 2020 Checklist

Additional file 2: Search strategies and Keywords used to search for article in the selected databases.

Additional file 3: Data extraction format

Additional file 4: Quality assessment

Additional file 5: Evidence Profile-GRADE CERQualR2

## Data Availability

All data extracted or analysed during this review are included in the publication of this systematic review article.

## Abbreviations

IOM: International Organization for Migration
GRADE-CERQual: Grading of Recommendations Assessment, Development and Evaluation-Confidence in Evidence from Reviews of Qualitative research
JBI: Joanna Briggs Institute
MMAT: Mixed Method Appraisal Tool
PICOS: Population, Interest, Comparison, Outcomes, and Study types
PRISMA: Preferred Reporting Items for Systematic Reviews and Meta-Analyses
SDGs: Sustainable Development Goals
