# Supplementary material for: Barriers and facilitators of access to maternity care for African-born women living in Australia: a meta-synthesis of qualitative evidence
Source: Syst Rev. 2024 Aug 9;13:215. doi: 10.1186/s13643-024-02628-8 (PMC11312702; doi:10.1186/s13643-024-02628-8)
Supplement: Supplementary file 2 — Additional file 2: Search strategies and Keywords used to search for article in the selected databases. [file 13643_2024_2628_MOESM2_ESM.docx]

**Additional File 1: Search strategies and Keywords used to search for article in the selected databases.**

Top of Form

| \| 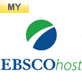 **MEDLINE Complete** \| Sunday, April 16, 2023, 11:47:18 AM \| \| --- \| --- \|   **Search modes[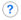](javascript:openWideTip('https://support.ebsco.com/help/?int=ehost&lang=en&feature_id=SrcMode&TOC_ID=Always&SI=0&BU=0&GU=1&PS=0&ver=live&dbs=%27))**   -  Boolean/Phrase |
| --- | --- | --- |

| **#** | **Query** | **Limiters/Expanders** | **Last Run Via** | **Results** |
| --- | --- | --- | --- | --- |
| S16 | S1 AND S12 AND S13 AND S14 AND S15 | Search modes - Boolean/Phrase | Interface - EBSCOhost Research Databases Search Screen - Advanced Search Database - MEDLINE Complete | 203 |
| S15 | S8 OR S9 OR S10 OR S11 | Search modes - Boolean/Phrase | Interface - EBSCOhost Research Databases Search Screen - Advanced Search Database - MEDLINE Complete | 13,720,389 |
| S14 | S2 OR S3 OR S4 OR S5 OR S6 OR S7 | Search modes - Boolean/Phrase | Interface - EBSCOhost Research Databases Search Screen - Advanced Search Database - MEDLINE Complete | 11,046,658 |
| S13 | Australia* | Search modes - Boolean/Phrase | Interface - EBSCOhost Research Databases Search Screen - Advanced Search Database - MEDLINE Complete | 948,734 |
| S12 | Africa* | Search modes - Boolean/Phrase | Interface - EBSCOhost Research Databases Search Screen - Advanced Search Database - MEDLINE Complete | 523,741 |
| S11 | access* | Search modes - Boolean/Phrase | Interface - EBSCOhost Research Databases Search Screen - Advanced Search Database - MEDLINE Complete | 825,411 |
| S10 | utiliz* OR Utilis* or use | Search modes - Boolean/Phrase | Interface - EBSCOhost Research Databases Search Screen - Advanced Search Database - MEDLINE Complete | 6,622,359 |
| S9 | facilitat* OR enabler* or motivator* | Search modes - Boolean/Phrase | Interface - EBSCOhost Research Databases Search Screen - Advanced Search Database - MEDLINE Complete | 751,214 |
| S8 | barrier* or obstacle* or factor* or challenge* or determinant* or hinder* or impede* | Search modes - Boolean/Phrase | Interface - EBSCOhost Research Databases Search Screen - Advanced Search Database - MEDLINE Complete | 8,188,969 |
| S7 | “Postnatal care” or “postpartum care” | Search modes - Boolean/Phrase | Interface - EBSCOhost Research Databases Search Screen - Advanced Search Database - MEDLINE Complete | 9,902 |
| S6 | Delivery or birth or lab?r or childbirth | Search modes - Boolean/Phrase | Interface - EBSCOhost Research Databases Search Screen - Advanced Search Database - MEDLINE Complete | 1,292,669 |
| S5 | Pregnan* or “antenatal care” or “prenatal care” or “obstetric care” | Search modes - Boolean/Phrase | Interface - EBSCOhost Research Databases Search Screen - Advanced Search Database - MEDLINE Complete | 1,169,610 |
| S4 | “wom?n health” or “maternal health" or “maternal health servic*” or “maternal health care” OR “maternity care” | Search modes - Boolean/Phrase | Interface - EBSCOhost Research Databases Search Screen - Advanced Search Database - MEDLINE Complete | 36,906 |
| S3 | Mother* or motherhood or maternal | Search modes - Boolean/Phrase | Interface - EBSCOhost Research Databases Search Screen - Advanced Search Database - MEDLINE Complete | 632,064 |
| S2 | Wom?n or female* | Search modes - Boolean/Phrase | Interface - EBSCOhost Research Databases Search Screen - Advanced Search Database - MEDLINE Complete | 10,207,830 |
| S1 | Migrant OR migration OR immigrant OR refugee OR humanitarian or asylum seeker | Search modes - Boolean/Phrase | Interface - EBSCOhost Research Databases Search Screen - Advanced Search Database - MEDLINE Complete | 436,005 |

Bottom of Form

Top of Form

| 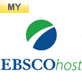 CINAHL Complete | Sunday, April 16, 2023, 12:25:12 PM |
| --- | --- |

**Search modes[
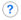
](javascript:openWideTip('https://support.ebsco.com/help/?int=ehost&lang=en&feature_id=SrcMode&TOC_ID=Always&SI=0&BU=0&GU=1&PS=0&ver=live&dbs=%27))**

- Boolean/Phrase

| **#** | **Query** | **Limiters/Expanders** | **Last Run Via** | **Results** |
| --- | --- | --- | --- | --- |
| S16 | S1 AND S12 AND S13 AND S14 AND S15 | Search modes - Boolean/Phrase | Interface - EBSCOhost Research Databases Search Screen - Advanced Search Database - CINAHL Complete | 157 |
| S15 | S8 OR S9 OR S10 OR S11 | Search modes - Boolean/Phrase | Interface - EBSCOhost Research Databases Search Screen - Advanced Search Database - CINAHL Complete | 3,429,489 |
| S14 | S2 OR S3 OR S4 OR S5 OR S6 OR S7 | Search modes - Boolean/Phrase | Interface - EBSCOhost Research Databases Search Screen - Advanced Search Database - CINAHL Complete | 2,713,784 |
| S13 | Australia* | Search modes - Boolean/Phrase | Interface - EBSCOhost Research Databases Search Screen - Advanced Search Database - CINAHL Complete | 138,201 |
| S12 | Africa* | Search modes - Boolean/Phrase | Interface - EBSCOhost Research Databases Search Screen - Advanced Search Database - CINAHL Complete | 102,847 |
| S11 | access* | Search modes - Boolean/Phrase | Interface - EBSCOhost Research Databases Search Screen - Advanced Search Database - CINAHL Complete | 305,359 |
| S10 | utiliz* OR Utilis* or use | Search modes - Boolean/Phrase | Interface - EBSCOhost Research Databases Search Screen - Advanced Search Database - CINAHL Complete | 1,656,222 |
| S9 | facilitat* OR enabler* or motivator* | Search modes - Boolean/Phrase | Interface - EBSCOhost Research Databases Search Screen - Advanced Search Database - CINAHL Complete | 154,232 |
| S8 | barrier* or obstacle* or factor* or challenge* or determinant* or hinder* or impede* | Search modes - Boolean/Phrase | Interface - EBSCOhost Research Databases Search Screen - Advanced Search Database - CINAHL Complete | 2,183,223 |
| S7 | “Postnatal care” or “postpartum care” | Search modes - Boolean/Phrase | Interface - EBSCOhost Research Databases Search Screen - Advanced Search Database - CINAHL Complete | 8,055 |
| S6 | Delivery or birth or lab?r or childbirth | Search modes - Boolean/Phrase | Interface - EBSCOhost Research Databases Search Screen - Advanced Search Database - CINAHL Complete | 409,721 |
| S5 | Pregnan* or “antenatal care” or “prenatal care” or “obstetric care” | Search modes - Boolean/Phrase | Interface - EBSCOhost Research Databases Search Screen - Advanced Search Database - CINAHL Complete | 298,794 |
| S4 | " wom?n health” or “maternal health" or “maternal health servic*” or “maternal health care” OR “maternity care” | Search modes - Boolean/Phrase | Interface - EBSCOhost Research Databases Search Screen - Advanced Search Database - CINAHL Complete | 19,067 |
| S3 | Mother* or motherhood or maternal | Search modes - Boolean/Phrase | Interface - EBSCOhost Research Databases Search Screen - Advanced Search Database - CINAHL Complete | 209,692 |
| S2 | Wom?n or female* | Search modes - Boolean/Phrase | Interface - EBSCOhost Research Databases Search Screen - Advanced Search Database - CINAHL Complete | 2,425,947 |
| S1 | Migrant OR migration OR immigrant OR refugee OR humanitarian or asylum seeker | Search modes - Boolean/Phrase | Interface - EBSCOhost Research Databases Search Screen - Advanced Search Database - CINAHL Complete | 73,257 |

Bottom of Form

Top of Form

| \| 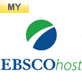 APA PsycInfo Sunday, April 16, 2023, 2:36:43 PM \|  \| \| --- \| --- \|   **Search modes[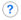](javascript:openWideTip('https://support.ebsco.com/help/?int=ehost&lang=en&feature_id=SrcMode&TOC_ID=Always&SI=0&BU=0&GU=1&PS=0&ver=live&dbs=%27))**   -  Boolean/Phrase |
| --- | --- | --- |

| **#** | **Query** | **Limiters/Expanders** | **Last Run Via** | **Results** |
| --- | --- | --- | --- | --- |
| S16 | S1 AND S12 AND S13 AND S14 AND S15 | Search modes - Boolean/Phrase | Interface - EBSCOhost Research Databases Search Screen - Advanced Search Database - APA PsycInfo | 91 |
| S15 | S8 OR S9 OR S10 OR S11 | Search modes - Boolean/Phrase | Interface - EBSCOhost Research Databases Search Screen - Advanced Search Database - APA PsycInfo | 2,453,938 |
| S14 | S2 OR S3 OR S4 OR S5 OR S6 OR S7 | Search modes - Boolean/Phrase | Interface - EBSCOhost Research Databases Search Screen - Advanced Search Database - APA PsycInfo | 1,553,732 |
| S13 | Australia* | Search modes - Boolean/Phrase | Interface - EBSCOhost Research Databases Search Screen - Advanced Search Database - APA PsycInfo | 229,123 |
| S12 | Africa* | Search modes - Boolean/Phrase | Interface - EBSCOhost Research Databases Search Screen - Advanced Search Database - APA PsycInfo | 121,323 |
| S11 | access* | Search modes - Boolean/Phrase | Interface - EBSCOhost Research Databases Search Screen - Advanced Search Database - APA PsycInfo | 199,061 |
| S10 | utiliz* OR Utilis* or use | Search modes - Boolean/Phrase | Interface - EBSCOhost Research Databases Search Screen - Advanced Search Database - APA PsycInfo | 1,120,293 |
| S9 | facilitat* OR enabler* or motivator* | Search modes - Boolean/Phrase | Interface - EBSCOhost Research Databases Search Screen - Advanced Search Database - APA PsycInfo | 212,246 |
| S8 | barrier* or obstacle* or factor* or challenge* or determinant* or hinder* or impede* | Search modes - Boolean/Phrase | Interface - EBSCOhost Research Databases Search Screen - Advanced Search Database - APA PsycInfo | 1,515,754 |
| S7 | “Postnatal care” or “postpartum care” | Search modes - Boolean/Phrase | Interface - EBSCOhost Research Databases Search Screen - Advanced Search Database - APA PsycInfo | 1,282 |
| S6 | Delivery or birth or lab?r or childbirth | Search modes - Boolean/Phrase | Interface - EBSCOhost Research Databases Search Screen - Advanced Search Database - APA PsycInfo | 209,910 |
| S5 | Pregnan* or “antenatal care” or “prenatal care” or “obstetric care” | Search modes - Boolean/Phrase | Interface - EBSCOhost Research Databases Search Screen - Advanced Search Database - APA PsycInfo | 77,991 |
| S4 | “wom?n health” or “maternal health" or “maternal health servic*” or “maternal health care” OR “maternity care” | Search modes - Boolean/Phrase | Interface - EBSCOhost Research Databases Search Screen - Advanced Search Database - APA PsycInfo | 6,326 |
| S3 | Mother* or motherhood or maternal | Search modes - Boolean/Phrase | Interface - EBSCOhost Research Databases Search Screen - Advanced Search Database - APA PsycInfo | 188,846 |
| S2 | Wom?n or female* | Search modes - Boolean/Phrase | Interface - EBSCOhost Research Databases Search Screen - Advanced Search Database - APA PsycInfo | 1,326,982 |
| S1 | Migrant OR migration OR immigrant OR refugee OR humanitarian or asylum seeker | Search modes - Boolean/Phrase | Interface - EBSCOhost Research Databases Search Screen - Advanced Search Database - APA PsycInfo | 74,196 |

Bottom of Form

**
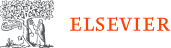
 Embase**

|  | **Query** | **Database** | **Last results** | **Last updated** | **Created** |
| --- | --- | --- | --- | --- | --- |
| #16 | #1 AND #2 AND #13 AND #14 AND #15 | Embase | 224 | 2023-04-16 | 2023-04-16 |
| #15 | #7 OR #8 OR #9 OR #10 OR #11 OR #12 | Embase | 13,459,767 | 2023-04-16 | 2023-04-16 |
| #14 | #3 OR #46OR #5 OR #6 | Embase | 13,777,234 | 2023-04-16 | 2023-04-16 |
| #13 | 'Australia'/exp OR Australia$ | Embase | 1,544,386 | 2023-04-16 | 2023-04-16 |
| #12 | 'Africa'/exp OR Africa$ | Embase | 885,938 | 2023-04-16 | 2023-04-16 |
| #11 | 'access'/exp OR access$ | Embase | 943,485 | 2023-04-16 | 2023-04-16 |
| #10 | 'utilize' OR utilize$ OR 'utilise' OR utilise$ OR 'use' OR use | Embase | 5,443,963 | 2023-04-16 | 2023-04-16 |
| #9 | 'facilitate' OR facilitate$ OR 'enabler' OR enabler$ OR 'motivator' OR motivator$ | Embase | 729,982 | 2023-04-16 | 2023-04-16 |
| #8 | 'barrier'/exp OR barrier$ OR 'obstacle' OR obstacle$ OR 'factor' OR factor$ OR 'challenge'/exp OR challenge$ OR 'determinant'/exp OR determinant$ OR 'hinder' OR hinder$ OR 'impede' OR impede$ | Embase | 9,060,368 | 2023-04-16 | 2023-04-16 |
| #7 | 'postnatal care'/exp OR 'postpartum care'/exp | Embase | 145,560 | 2023-04-16 | 2023-04-16 |
| #6 | 'delivery'/exp OR 'birth'/exp OR 'lab?r' OR 'childbirth'/exp | Embase | 445,855 | 2023-04-16 | 2023-04-16 |
| #5 | 'pregnan' OR pregnan$ OR 'antenatal care'/exp OR 'prenatal care'/exp OR 'obstetri9care'/exp | Embase | 856,917 | 2023-04-16 | 2023-04-16 |
| #4 | 'wom?n health' OR 'maternal health'/exp OR 'maternal health service'/exp OR 'maternal health care'/exp OR 'maternity care'/exp | Embase | 68,657 | 2023-04-16 | 2023-04-16 |
| #3 | 'mother'/exp OR mother$ OR 'motherhood'/exp OR 'maternal'/exp | Embase | 438,370 | 2023-04-16 | 2023-04-16 |
| #2 | 'wom?n' OR 'female'/exp OR female$ | Embase | 13,114,032 | 2023-04-16 | 2023-04-16 |
| #1 | 'migrant'/exp OR 'immigrant'/exp OR 'refugee'/exp OR 'humanitarian'/exp OR 'asylum seeker'/exp | Embase | 52,803 | 2023-04-16 | 2023-04-16 |


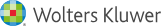
 Ovid®

Top of Form

Database(s): **Maternity & Infant Care Database (MIDIRS)**1971 to February 13, 2024
Search Strategy:

| **#** | **Searches** | **Results** |
| --- | --- | --- |
| 16 | 1 AND 13 AND 14 AND 15 AND 16 | 63 |
| 15 | 8 OR 9 OR 10 OR 11 OR 12 | 13606 |
| 14 | 2 OR 3 OR 4 OR 5 OR 6 OR7 | 12284 |
| 13 | Australia {Including Limited Related Terms} | 7876 |
| 12 | Africa {Including Limited Related Terms} | 10965 |
| 11 | access {Including Limited Related Terms} | 12521 |
| 10 | utilization or utilisation or utilise or utilize or use {Including Limited Related Terms} | 4002 |
| 9 | facilitators or enablers or motivators {Including Limited Related Terms} | 222 |
| 8 | barriers or obstacles or factors or challenges or determinants or hinderers or impeders {Including Limited Related Terms} | 1006 |
| 7 | "Postnatal care" or "postpartum care" {Including Limited Related Terms} | 5754 |
| 6 | Delivery or birth or labour or childbirth {Including Limited Related Terms} | 10696 |
| 5 | "Pregnancy care" or "antenatal care" or "prenatal care" or "obstetric care" {Including Limited Related Terms} | 7429 |
| 4 | "women's health" or "maternal health" or "maternal health services" or "maternal health care" OR "maternity care" {Including Limited Related Terms} | 1451 |
| 3 | Mother or mothers or motherhood or maternal {Including Limited Related Terms} | 11663 |
| 2 | Woman or women or female or females {Including Limited Related Terms} | 11428 |
| 1 | migration OR immigrant OR refugee OR humanitarian or asylum seeker {Including Limited Related Terms} | 266 |

* is a truncation symbol to retrieve terms with a common root within Medline, CINHAL, and PsychInfo.

$ is a truncation symbol to retrieve terms with a common root within EMBASE.
