# Supplementary material for: Barriers and facilitators of access to maternity care for African-born women living in Australia: a meta-synthesis of qualitative evidence
Source: Syst Rev. 2024 Aug 9;13:215. doi: 10.1186/s13643-024-02628-8 (PMC11312702; doi:10.1186/s13643-024-02628-8)
Supplement: Supplementary file 4 — Additional file 4: Quality assessment [file 13643_2024_2628_MOESM4_ESM.docx]

**Quality of the included papers, Mixed Methods Appraisal Tool (MMAT), version 2018**

| **Study designs** | Studies | **Methodological quality criteria** | **Responses** | | | |
| --- | --- | --- | --- | --- | --- | --- |
|  |  |  | Yes | No | Can’t tell | Comments |
| 1. Qualitative | Benza 2017 | S1. Are there clear research questions? | x |  |  |  |
|  |  | S2. Do the collected data allow to address the research questions? | x |  |  |  |
|  |  | 1.1. Is the qualitative approach appropriate to answer the research question? | 1 |  |  |  |
|  |  | 1.2. Are the qualitative data collection methods adequate to address the research question? | 1 |  |  |  |
|  |  | 1.3. Are the findings adequately derived from the data? | 1 |  |  |  |
|  |  | 1.4. Is the interpretation of results sufficiently substantiated by data? | 1 |  |  |  |
|  |  | 1.5. Is there coherence between qualitative data sources, collection, analysis and interpretation? | 1 |  |  |  |
|  |  | **Total item score** | 5 |  |  |  |
|  | Carolan 2007 | S1. Are there clear research questions? | x |  |  |  |
|  |  | S2. Do the collected data allow to address the research questions? | x |  |  |  |
|  |  | 1.1. Is the qualitative approach appropriate to answer the research question? | 1 |  |  |  |
|  |  | 1.2. Are the qualitative data collection methods adequate to address the research question? | 1 |  |  |  |
|  |  | 1.3. Are the findings adequately derived from the data? | 1 |  |  |  |
|  |  | 1.4. Is the interpretation of results sufficiently substantiated by data? | 1 |  |  |  |
|  |  | 1.5. Is there coherence between qualitative data sources, collection, analysis and interpretation? | 1 |  |  |  |
|  |  | **Total item score** | 5 |  |  |  |
|  | Carolan 2010 | S1. Are there clear research questions? | x |  |  |  |
|  |  | S2. Do the collected data allow to address the research questions? | x |  |  |  |
|  |  | 1.1. Is the qualitative approach appropriate to answer the research question? | 1 |  |  |  |
|  |  | 1.2. Are the qualitative data collection methods adequate to address the research question? | 1 |  |  |  |
|  |  | 1.3. Are the findings adequately derived from the data? | 1 |  |  |  |
|  |  | 1.4. Is the interpretation of results sufficiently substantiated by data? | 1 |  |  |  |
|  |  | 1.5. Is there coherence between qualitative data sources, collection, analysis and interpretation? | 1 |  |  |  |
|  |  | **Total item score** | 5 |  |  |  |
|  | Due 2022 | S1. Are there clear research questions? | x |  |  |  |
|  |  | S2. Do the collected data allow to address the research questions? | x |  |  |  |
|  |  | 1.1. Is the qualitative approach appropriate to answer the research question? | 1 |  |  |  |
|  |  | 1.2. Are the qualitative data collection methods adequate to address the research question? | 1 |  |  |  |
|  |  | 1.3. Are the findings adequately derived from the data? | 1 |  |  |  |
|  |  | 1.4. Is the interpretation of results sufficiently substantiated by data? | 1 |  |  |  |
|  |  | 1.5. Is there coherence between qualitative data sources, collection, analysis and interpretation? | 1 |  |  |  |
|  |  | **Total item score** | 5 |  |  |  |
|  | Hawkey 2022 | S1. Are there clear research questions? | x |  |  |  |
|  |  | S2. Do the collected data allow to address the research questions? | x |  |  |  |
|  |  | 1.1. Is the qualitative approach appropriate to answer the research question? | 1 |  |  |  |
|  |  | 1.2. Are the qualitative data collection methods adequate to address the research question? | 1 |  |  |  |
|  |  | 1.3. Are the findings adequately derived from the data? | 1 |  |  |  |
|  |  | 1.4. Is the interpretation of results sufficiently substantiated by data? | 1 |  |  |  |
|  |  | 1.5. Is there coherence between qualitative data sources, collection, analysis and interpretation? | 1 |  |  |  |
|  |  | **Total item score** | 5 |  |  |  |
|  | Manderson 2003 | S1. Are there clear research questions? | x |  |  |  |
|  |  | S2. Do the collected data allow to address the research questions? | x |  |  |  |
|  |  | 1.1. Is the qualitative approach appropriate to answer the research question? | 1 |  |  |  |
|  |  | 1.2. Are the qualitative data collection methods adequate to address the research question? | 1 |  |  |  |
|  |  | 1.3. Are the findings adequately derived from the data? | 1 |  |  |  |
|  |  | 1.4. Is the interpretation of results sufficiently substantiated by data? |  | 1 |  |  |
|  |  | 1.5. Is there coherence between qualitative data sources, collection, analysis and interpretation? | 1 |  |  |  |
|  |  | **Total item score** | **4** | **1** |  |  |
|  | Mohale 2017 | S1. Are there clear research questions? | x |  |  |  |
|  |  | S2. Do the collected data allow to address the research questions? | x |  |  |  |
|  |  | 1.1. Is the qualitative approach appropriate to answer the research question? | 1 |  |  |  |
|  |  | 1.2. Are the qualitative data collection methods adequate to address the research question? | 1 |  |  |  |
|  |  | 1.3. Are the findings adequately derived from the data? | 1 |  |  |  |
|  |  | 1.4. Is the interpretation of results sufficiently substantiated by data? | 1 |  |  |  |
|  |  | 1.5. Is there coherence between qualitative data sources, collection, analysis and interpretation? | 1 |  |  |  |
|  |  | **Total item score** | 5 |  |  |  |
|  | Murray 2010  , | S1. Are there clear research questions? | x |  |  |  |
|  |  | S2. Do the collected data allow to address the research questions? | x |  |  |  |
|  |  | 1.1. Is the qualitative approach appropriate to answer the research question? | 1 |  |  |  |
|  |  | 1.2. Are the qualitative data collection methods adequate to address the research question? | 1 |  |  |  |
|  |  | 1.3. Are the findings adequately derived from the data? | 1 |  |  |  |
|  |  | 1.4. Is the interpretation of results sufficiently substantiated by data? |  | 1 |  |  |
|  |  | 1.5. Is there coherence between qualitative data sources, collection, analysis and interpretation? | 1 |  |  |  |
|  |  | **Total item score** | **4** | **1** |  |  |
|  | Owens 2016, | S1. Are there clear research questions? | x |  |  |  |
|  |  | S2. Do the collected data allow to address the research questions? | x |  |  |  |
|  |  | 1.1. Is the qualitative approach appropriate to answer the research question? | 1 |  |  |  |
|  |  | 1.2. Are the qualitative data collection methods adequate to address the research question? | 1 |  |  |  |
|  |  | 1.3. Are the findings adequately derived from the data? | 1 |  |  |  |
|  |  | 1.4. Is the interpretation of results sufficiently substantiated by data? | 1 |  |  |  |
|  |  | 1.5. Is there coherence between qualitative data sources, collection, analysis and interpretation? | 1 |  |  |  |
|  |  | **Total item score** | 5 |  |  |  |
|  | Tyler 2014 | S1. Are there clear research questions? | x |  |  |  |
|  |  | S2. Do the collected data allow to address the research questions? | x |  |  |  |
|  |  | 1.1. Is the qualitative approach appropriate to answer the research question? | 1 |  |  |  |
|  |  | 1.2. Are the qualitative data collection methods adequate to address the research question? | 1 |  |  |  |
|  |  | 1.3. Are the findings adequately derived from the data? | 1 |  |  |  |
|  |  | 1.4. Is the interpretation of results sufficiently substantiated by data? |  | 1 |  |  |
|  |  | 1.5. Is there coherence between qualitative data sources, collection, analysis and interpretation? | 1 |  |  |  |
|  |  | **Total item score** | **4** | **1** |  |  |
| 2. Mixed methods | Correa-Velez 2012 | S1. Are there clear research questions? | x |  |  |  |
|  |  | S2. Do the collected data allow to address the research questions? | x |  |  |  |
|  |  | 5.1. Is there an adequate rationale for using a mixed methods design to address the research question? | 1 |  |  |  |
|  |  | 5.2. Are the different components of the study effectively integrated to answer the research question? | 1 |  |  |  |
|  |  | 5.3. Are the outputs of the integration of qualitative and quantitative components adequately interpreted? | 1 |  |  |  |
|  |  | 5.4. Are divergences and inconsistencies between quantitative and qualitative results adequately addressed? |  | 1 |  |  |
|  |  | 5.5. Do the different components of the study adhere to the quality criteria of each tradition of the methods involved? | 1 |  |  |  |
|  |  | **Total item score** | **4** | **1** |  |  |
