# Supplementary material for: Barriers and facilitators of access to maternity care for African-born women living in Australia: a meta-synthesis of qualitative evidence
Source: Syst Rev. 2024 Aug 9;13:215. doi: 10.1186/s13643-024-02628-8 (PMC11312702; doi:10.1186/s13643-024-02628-8)
Supplement: Supplementary file 5 — Additional file 5: Evidence Profile-GRADE CERQualR2 [file 13643_2024_2628_MOESM5_ESM.docx]

**Additional File 5: Evidence Profile - GRADE CERQual**

| **Finding** | **Contributing records** | **Methodological limitations** | **Coherence** | **Adequacy** | **Relevance** | **Overall Confidence** |
| --- | --- | --- | --- | --- | --- | --- |
| **Analytical theme 1: Approachability and ability to perceive** | | | | | | |
| **Finding 1:** African-born women living in Australia lack awareness about the available maternity care options due to a shortage of information. | [47, 50] [40, 42] | **Minor concerns** | **No or very minor concerns** | **No or very minor concerns** | **No or very minor concerns** | **High** |
| **Finding 2:** A lack of familiarity with some medical interventions, negative attitude toward some medical technologies, misunderstanding of the purpose of the service, considering birth as a natural process, and a lack of knowledge about where to seek maternity care impacted African-born women’s access to care. | [42, 48] [46] [50] | **Moderate concerns** | **Minor concerns** | **Minor concerns** | **Minor concerns** | **Moderate** |
| **Finding 3:** African-born women have an interest in attending and receiving maternal health information provided in hospitals. | [43] [42] | **No or very minor concerns** | **Moderate concerns** | **Minor concerns** | **Minor concerns** | **Moderate** |
| **Analytical theme 2: Acceptability and ability to seek** | | | | | | |
| **Finding 4:** Most African-born women felt that the services they received were not culturally responsive and did not meet their religious needs. Most women claimed that their preferences for female service providers were not met. | [42, 44] [43, 46] [48] | **Minor concerns** | **Minor concerns** | **Minor concerns** | **Moderate concerns** | **Moderate** |
| **Finding 5:** Most African-born women felt being discriminated against, labelled, felt alone and different in the hospital environment, and received racially stereotypical comments in the service delivery facilities that affected access to care. | [40] [41, 48] | **Minor concerns** | **No or very minor concerns** | **No or very minor concerns** | **No or very minor concerns** | **High** |
| **Finding 6:** Prior maternity experiences such as home birth experience in home countries and negative maternity outcomes during previous pregnancies affected African-born women's access to maternity care. | [42] [44] | **No or very minor concerns** | **No or very minor concerns** | **Minor concerns** | **No or very minor concerns** | **High** |
| **Finding 7:** Access to maternity care was affected among African-born women who were asked inappropriate questions by midwives, those who perceived a lack of respect during care episodes, and those who considered service providers as rushing during consultation because women lost trust in service providers. | [48] [40] [45, 46] | **Minor concerns** | **Moderate concerns** | **Minor concerns** | **Minor concerns** | **Moderate** |
| **Finding 8:** A lack of social support such as a lack of family involvement during labour limited African-born women's access to maternity care. | [43, 45] | **Moderate concerns** | **Minor**  **concerns** | **No or very low concerns** | **Minor**  **concerns** | **Moderate** |
| **Finding 9:** African-born women who felt that staff had a positive attitude toward them, who considered midwives to be kind and respectful, and who were asked about their wellbeing by staff and those who felt being understood and valued by the service providers were motivated to access maternity care. | [41, 47] [48] [49] | **Minor concerns** | **Moderate concerns** | **Minor concerns** | **No or very minor**  **concerns** | **Moderate** |
| **Finding 10:** Cultural assimilation, perceiving their own culture was respected, and access to bicultural social workers assisted women in accepting the Australian maternity care system. | [42, 44] | **Minor concern** | **No or very minor**  **concerns** | No or very minor  concerns | **No or very minor**  **concerns** | **High** |
| **Analytical theme 3: Availability and accommodation, and ability to reach** | | | | | | |
| **Finding 11:** African-born women considered the maternity care system inaccessible because of difficulties navigating the health system, long waiting times, and difficulty accessing public transport. | [48] [43], [47] | **Minor concerns** | **Minor concerns** | **Minor**  **concerns** | **No or very minor**  **concerns** | **Moderate** |
| **Analytical theme 4: Affordability and ability to pay** | | | | | | |
| **Finding 12:** African-born women prioritised resettlement over receiving healthcare. Compounded with other commitments such as childcare roles that resulted in the loss of jobs, this created financial constraints to seek maternity care. | [42] [43] | **Minor concerns**  **concerns** | **No or very minor**  **concerns** | **No or very minor**  **concerns** | **No or very minor** | **High** |
| **Finding 13:** African-born women perceived that multiple maternity services including education classes and various pain killers are available in a service delivery point and facilities are close to their homes. These facilitated access to maternity care. | [47] [48] [41, 49] | **Minor concerns** | **No or very minor concerns** | **Moderate concerns** | **Minor concerns** | **Moderate** |
| **Analytical theme 5: Appropriateness and ability to engage** | | | | | | |
| **Finding 14:** Language barriers and problems related to interpreter services such as the gender of the interpreter, late arrival, fear of confidentiality breach, and lack of awareness about the availability of interpreter services affected African-born women's access to maternity care. | [42, 47, 48, 50] [43, 47, 48] | **Minor concerns** | **Very minor concerns** | **Very minor concerns** | **Minor concerns** | **High** |
| **Finding 15:** Due to the inadequacy of continuity of care or a lack of awareness about continuity of care, African-born women were frustrated when attended to by multiple midwives at different appointments. | [44, 48] [47] | **Minor concerns** | **Minor concerns** | **Moderate concerns** | **Minor concerns** | **Moderate** |
| **Finding 16:** Midwives lacked experience in providing care for African-born women with circumcision, ignored women’s requests, and failed to recognise women’s prior pregnancy experiences. Thus African-born women perceived the quality of maternity care as suboptimal, which impacted their care-seeking behaviour. | [44] [43, 48] | **Minor concerns** | **No or very minor concerns** | **No or very minor concerns** | **Minor concerns** | **High** |
| **Finding 17:** African-born women were frustrated when they were not requested consent for the presence of students during labour, when they were not explained the services they received, and when they were not given the opportunity to have control over their care. | [44] [46] | **Minor concerns** | **No or very minor concerns** | **Minor concerns** | **Minor concerns** | **Moderate** |
| **Finding 18:** African-born women who lived in Australia for a long time had the opportunity to learn English and this created a sense of empowerment. These women have positive attitudes about healthcare as they feel they were being accepted and supported by midwives. | [48][42, 49] | **Minor concerns** | **No or very minor concerns** | **No or very minor concerns** | **No or very minor concerns** | **High** |
